# Intermediate Filaments as Organizers of Cellular Space: How They Affect Mitochondrial Structure and Function

**DOI:** 10.3390/cells5030030

**Published:** 2016-07-05

**Authors:** Nicole Schwarz, Rudolf E. Leube

**Affiliations:** Institute of Molecular and Cellular Anatomy, RWTH Aachen University, Wendlingweg 2, Aachen 52074, Germany; rleube@ukaachen.de

**Keywords:** mitochondrion, mitochondria-associated membrane, intermediate filament, neurofilament, desmin, keratin, vimentin

## Abstract

Intermediate filaments together with actin filaments and microtubules form the cytoskeleton, which is a complex and highly dynamic 3D network. Intermediate filaments are the major mechanical stress protectors but also affect cell growth, differentiation, signal transduction, and migration. Using intermediate filament-mitochondrial crosstalk as a prominent example, this review emphasizes the importance of intermediate filaments as crucial organizers of cytoplasmic space to support these functions. We summarize observations in different mammalian cell types which demonstrate how intermediate filaments influence mitochondrial morphology, subcellular localization, and function through direct and indirect interactions and how perturbations of these interactions may lead to human diseases.

## 1. Introduction

In this review we will summarize current knowledge on the interactions between intermediate filaments and mitochondria in different tissues. This topic has been subject of multiple publications in the recent past but has not been systematically reviewed since 2005 [1].

### 1.1. Intermediate Filaments as Organizers of Cytoplasmic Space

Intermediate filaments are part of the cytoskeleton along with microtubules and actin filaments. They form three-dimensional scaffolds that are arranged in context-dependent networks to provide mechanical resilience. Intermediate filaments can be divided into cytoplasmic intermediate filaments and nuclear lamins. The constitutive intermediate filament proteins are expressed in a cell type- and differentiation-specific manner [2]. Based on their sequence homology, encoding gene structure, charge, assembly mechanism, and expression pattern the following types can be distinguished: epithelial type I and type II keratins; the type III intermediate filament proteins including vimentin, typically expressed in cells of mesenchymal origin, glial fibrillary acidic protein (GFAP) of glial cells, peripherin expressed mainly in neurons of the peripheral nervous system and desmin, which is found in muscle cells; the type IV intermediate filament proteins of neurons such as the three neurofilament proteins; the nuclear type V lamins; and the unusual type VI intermediate filament proteins phakinin and filensin, which form beaded filaments in the lens [2,3,4,5,6].

**Figure 1:**
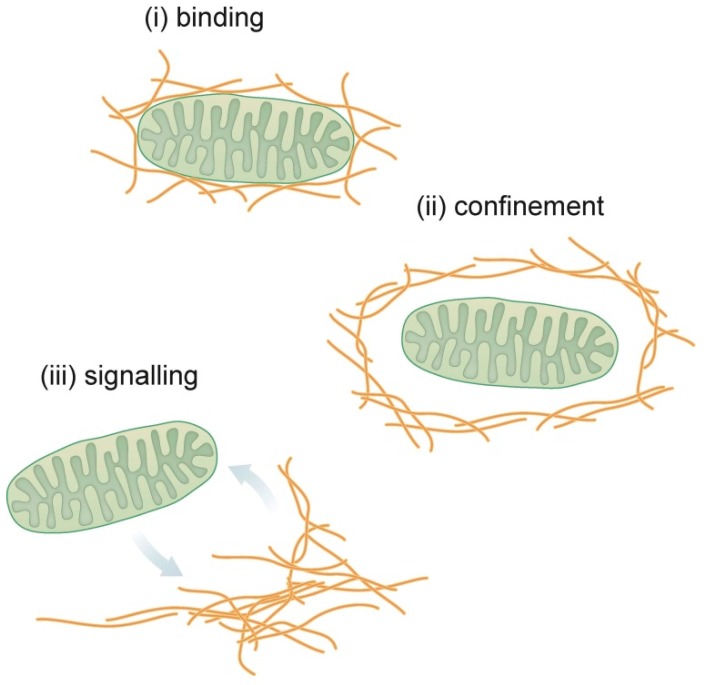
Schematic representation of different modes of interaction between intermediate filaments and mitochondria.

In addition to the established role of intermediate filaments as major mechanical integrators of cells and tissues, a variety of novel functions have been identified. These include regulation of apoptosis and cell growth, differentiation, signal transduction, and migration. The emerging view is that these functions are a consequence of intermediate filaments acting as organizers of cytoplasmic space. In accordance, intermediate filaments have been shown to determine nuclear shape and to affect the positioning of the Golgi apparatus, mitochondria, transport vesicles, lysosomes, melanosomes, centrosomes, ribosomes, lipid droplets, and junctions between cells and to the extracellular matrix [7,8,9,10,11,12,13,14,15,16]. Very little is known about the mechanistic and molecular nature of these interactions and how their perturbation contributes to the phenotypes of more than 80 human diseases that are caused by mutations in intermediate filament genes [17]. The scheme in Figure 1 portrays three major scenarios: (*i*) binding of intermediate filaments to cellular components through molecular linkage; (*ii*) confinement of cellular components by intermediate filament networks; and (*iii*) bidirectional signaling between cellular components and intermediate filaments.

### 1.2. Mitochondrial Trafficking and Shape Changes

Constant ATP supply is essential for virtually all cellular functions. In order to achieve local ATP production, mitochondria accumulate at regions of high-energy demand. Additionally, stationary mitochondria serve as calcium buffers to avoid harmful intracellular calcium spikes. Therefore, not only directed transport but also docking and anchoring mechanisms are needed to achieve the correct distribution of mitochondria. In the past, the contribution of actin filaments and microtubules to mitochondrial transport has been extensively examined [18,19]. For example, mitochondrial movement has been shown to depend on microtubule-associated motor proteins: plus-end-directed kinesin superfamily proteins (KIFs) and minus-end-directed cytoplasmic dynein [20]. Mitochondria attach to motor proteins via motor adaptors (TRAK1/2 or Milton) that are linked to mitochondrial membrane anchors (RhoT1/2 or Miro) [21,22]. Since intermediate filaments are intrinsically apolar they cannot provide directional cues to mitochondrial transport [23].

Immobilized mitochondria are likely anchored to the cytoskeleton. For both, microtubules and actin, mitochondrial docking mechanisms have been described that include mitochondrial membrane anchoring receptors. Anchoring of mitochondria to microtubules via the outer membrane protein syntaphilin arrests them and is regulated by the dynein light chain LC8 [24,25]. In *Drosophila*, tethering of mitochondria to the actin cytoskeleton reduces their motility and involves the association of myosin V and VI [26]. An increasing number of reports show that intermediate filaments also influence mitochondrial motility that will be discussed in more detail below.

In addition to mitochondrial motility, the mitochondrial shape is critical for mitochondrial function and seems to be influenced by intermediate filament proteins [1,27]. Mitochondrial morphology is determined by the balance between fusion and fission events. The key determinant of mitochondrial fission is the cytoplasmic GTPase Drp1 that can be recruited to mitochondria by several outer mitochondrial membrane proteins such as Fis1 and Mff1 [28,29,30]. Fusion events are mediated by the outer mitochondrial membrane proteins mitofusin 1 and 2 (Mfn1/2) and the inner mitochondrial membrane protein OPA1 [31,32]. Mitochondrial morphology changes in response to alterations in energy demand and nutrient availability. While elongated mitochondria are associated with a sustained and more-efficient ATP production, fragmented mitochondria produce less ATP [33].

## 2. Interactions of Intermediate Filaments and Mitochondria in Different Cell Types

Almost 40 years ago, several groups described a close spatial association between intermediate filaments and mitochondria in multiple cell types based on electron microscopy studies [34,35,36,37]. It was suggested that the intermediate filament network can serve as an anchorage site for mitochondria [38]. More generally, it was proposed that organelle distribution requires intermediate filaments [39]. Since then, many reports provided further insight how intermediate filaments contribute to mitochondrial localization and function in different cell types (summarized in Table 1).

### 2.1. Lessons Learned from Neurons: Intermediate Filaments Determine Mitochondrial Motility

Neurofilaments are the major intermediate filament proteins in neuronal cells. They are divided into light (NF-L), medium (NF-M), and heavy (NF-H) neurofilament proteins. NF-L associates with either NF-M or NF-H into the 10–12 nm intermediate filaments [50]. Positioning of mitochondria is especially important in axons where they produce not only ATP but also buffer Ca^2+^ to regulate neurotransmission in associated nerve terminals [51]. Axonal mitochondria can either be transported via microtubules or actin filaments. Only cells lacking both cytoskeletal components show complete mitochondrial arrest [18].

Interestingly, injection of excess neurofilament proteins into cultured neurons also leads to mitochondrial arrest [52]. It was not shown, however, whether this effect is specific to either neurofilament polypeptide. It was later demonstrated that mitochondrial distribution was not affected in neurons of mice expressing a tail-less NF-H mutant, while the full length NF-H induced perinuclear aggregation of mitochondria upon overexpression in cultivated cells [53,54]. The mutant NF-H lacks the highly phosphorylated end domain that is known to form extended sidearms that are involved in axonal intermediate filament arrangement [55,56]. Furthermore, it was shown that this domain of NF-H and also of NF-M competes with microtubule-associated protein 2 (MAP2) for binding to the voltage-dependent anion channel (VDAC) in the outer mitochondrial membrane and may thus affect the mitochondrial membrane potential [57]. Of note, stationary mitochondria have a higher membrane potential than motile mitochondria leading to a higher metabolic activity [58]. Furthermore, stationary mitochondria with higher membrane potential show increased binding to phosphorylated NF-L [59]. Taken together, the experimental findings support the notion that neurofilament binding to mitochondria affects their activity which appears to be linked to reduced mitochondrial mobility. Furthermore, NF-L knock-out mice display not only an increased mitochondrial motility with a reduced frequency of stops but also decreased overall length and fusion rate of mitochondria [40,41]. Overexpression of peripherin in a NF-L deficient background in turn results in a net retrograde transport of mitochondria, probably contributing to the pathology of neurodegenerative disorders with intermediate filament aggregate formation [41].

Moreover, mutations in NF-L lead to a misassembly of neurofilaments and cause devastating Charcot-Marie-Tooth (CMT) neuronal muscular dystrophy disease types 1 and 2 that are characterized by degeneration of long peripheral axons. Mutant NF-L was shown to perturb axonal transport of mitochondria, resulting in accumulation of mitochondria in cell bodies and subsequent impairment of energy supply in distal regions of axons [60]. Interestingly, most of the CMT type 2A cases are caused by mutations of Mfn2 leading to reduced fusion as well as a decline in mitochondrial motility [61,62]. The decrease is explained by the interaction of Mfn2 with the Miro/Milton complex, which has been implicated in the regulation of mitochondrial transport [62]. These findings suggest that NF-L is upstream of Mfn2 and may exert its disease-causing function through perturbed Mfn2 activity.

### 2.2. Lessons Learned from Mesenchymal Cells: Intermediate Filaments Bind to Mitochondria

The intermediate filament protein vimentin is mainly expressed in cells of mesenchymal origin. Vimentin has been reported to co-localize with mitochondria in several different cell lines [44,63,64,65]. Similar to neurofilaments, vimentin filaments are able to modulate the motile behavior and shape of mitochondria [66].

Intermediate filaments might either bind directly to mitochondria or via intermediate filament-associated proteins. One of the most likely bridging molecules is plectin, a large cytolinker protein that is ubiquitously expressed [67]. Its isoform 1b was shown to mediate vimentin linkage to mitochondria [68]. Plectin 1b knock-out resulted in mitochondrial shape changes with increased elongation but did not affect mitochondrial motility [69]. The latter shows that only some intermediate filament-dependent mitochondrial functions are mediated through plectin.

Interestingly, direct plectin-independent mitochondrial binding was demonstrated for vimentin. This binding is restricted to mitochondria with functional respiratory chain activity [43]. The binding activity was localized to the non-α-helical N-terminal domain of vimentin [42]. This domain meets the requirements for sequences targeted to the outer mitochondrial membrane [70]. As described above, mitochondrial anchoring sites are needed to ensure localized ATP production. Vimentin binding results in arrested mitochondria [42]. Of note, stationary mitochondria possess a higher membrane potential than motile mitochondria. Yet, reducing mitochondrial motility by disruption of actin filaments or microtubules does not change the membrane potential [71]. On the other hand, vimentin-null cells display not only more motile mitochondria but also mitochondria with lower membrane potential, which could be reversed by re-introducing vimentin [71]. Thus, the mitochondrial membrane potential may be regulated by the interaction of the N-terminal domain of vimentin with a still unknown receptor in the outer mitochondrial membrane [43]. The resulting elevation in the mitochondrial membrane potential then leads to enhanced ATP production.

A possible regulatory mechanism of the vimentin-mitochondrial interaction is suggested by findings of the Minin laboratory implicating the GTPase Rac1. They showed that activation of Rac1 induces phosphorylation of vimentin either directly or indirectly via PAK1 [72]. Phosphorylation of vimentin at the mitochondrial binding site, which lies in the most extensively phosphorylated vimentin subdomain, may thereby release motile mitochondria with a decreased membrane potential resulting in reduced ATP production [73].

The pathological relevance of vimentin for mitochondrial motility was recently elucidated in fibroblasts that were derived from patients carrying a mutation in gigaxonin, an E3 ligase adapter that targets intermediate filament proteins for degradation. The vimentin network is collapsed into cytoplasmic aggregates in these cells, leading to the accumulation of mitochondria near the vimentin-containing aggregates. In addition, mitochondrial motility is considerably reduced [74]. The mechanisms by which gigaxonin mutations in gigaxonin lead to these profound effects are not understood.

### 2.3. Lessons Learned from Muscle Cells: Intermediate Filaments Affect Mitochondrial Calcium Handling and Energy Production

The major intermediate filament protein in muscle cells is desmin. The three-dimensional filamentous desmin cytoskeleton provides highly flexible links between myofibrils and connects them to other intracellular components and the extracellular matrix through specialized contact sites [75]. Ablation of desmin leads to defects in the architecture of the contractile apparatus resulting in myopathy [76]. Desmin intermediate filaments are tethered to the nucleus and mitochondria to maintain their subcellular localization during and after contraction [77]. Desmin null mice helped to further delineate the relationship between intermediate filaments and mitochondria [15,45,78]. The nuclear as well as mitochondrial morphology and localization are disturbed in the absence of desmin [45,79]. Mitochondria of cardiac and skeletal muscle cells showed reduced cristae density along with vacuolization of the mitochondrial matrix and formation of giant mitochondria. Furthermore, extensive mitochondrial proliferation was observed in muscle cells of desmin null mice, especially after work overload [45]. The described mitochondrial phenotypes are recapitulated in patients harboring desmin mutations [80,81].

Altered spatial organization of mitochondria goes along with altered distance to the endo(sarco)plasmic reticulum (ER). Close contacts between mitochondria and ER are referred to as mitochondria-associated membranes (MAMs) [82]. They are involved in lipid traffic between both organelles and in transfer of Ca^2+^ from the ER to mitochondria through a complex formed by the voltage-dependent anion channel (VDAC) and inositol 1,4,5-trisphophate receptors (IP3Rs) [83,84,85]. Proteomic analysis of mitochondria isolated from heart tissue has shown that several key pathways, including apoptosis, calcium homeostasis, and different metabolic pathways are perturbed in desmin knock-out mice. One of the differentially expressed proteins is VDAC, suggesting a relationship between the desmin-determined structural organization of the cell and mitochondrial functions [86]. In accordance, transgenic mice expressing the aggregation-prone desmin mutant L345P displayed severe alterations of mitochondrial morphology and Ca^2+^ handling in skeletal and cardiac muscle [79]. Mitochondrial Ca^2+^ uptake was increased, whereas release was reduced resulting in mitochondrial Ca^2+^ overload ([79] for contradictory in vitro results see, however, [87]).

While a slight increase in mitochondrial Ca^2+^ increases ATP production, mitochondrial Ca^2+^ overload leads to autophagy and apoptosis [88,89]. Members of the Bcl-2 protein family are known to regulate apoptosis [90]. They bind to VDAC and thereby regulate mitochondrial membrane potential and cytochrome c release during apoptosis [91]. The anti-apoptotic Bcl-2 rendered mitochondria more resistant to exposure of high levels of calcium, while desmin knock-out rendered mitochondria more susceptible to high levels of calcium [92]. Bcl-2 expression prevents calcium induced swelling of mitochondria, subsequent cytochrome c release and hence apoptosis induction [93]. Consequently and quite remarkably, Bcl-2 overexpression in desmin null cardiomyocytes of transgenic mice rescued the mitochondrial defects and ameliorated the overall cardiomyopathy [94].

In mitochondria of desmin null heart, creatine kinase activity was elevated [95]. Creatine phosphate, an important cellular energy source, is produced by mitochondrial creatine kinase that resides in the mitochondrial intermembrane space to buffer high ATP levels in cells with high and fluctuating energy requirements. Creatine phosphate is processed by cytosolic creatine kinases to ensure sufficient ATP supply in situations of sudden energy demands. Mitochondrial creatine kinase is near to VDAC that is localized in the outer mitochondrial membrane and the adenine nucleotide translocator (ANT) of the inner mitochondrial membrane. The complex of the three proteins is essential for facilitating the transport of ATP from the mitochondrial matrix to the mitochondrial intermembrane space and, conversely, the transport of ADP from the mitochondrial intermembrane space into the mitochondrial matrix which is referred to as metabolite channeling or functional coupling [96]. In soleus muscle, desmin ablation leads to an uncoupling of mitochondrial creatine kinase and ANT [45]. As a consequence ADP is reduced in the mitochondrial matrix leading to a lower ADP-stimulated respiratory rate and, hence, reduced ATP production. Accordingly, the number of mitochondria with lower ADP-stimulated respiratory rates is increased in striated muscles of desmin knock-out animals resulting in an overall decrease in respiratory capacity [97].

### 2.4. Lessons Learned in Epithelia: Intermediate Filaments Affect Mitochondrial Lipid Metabolism and Communicate through Signaling with Mitochondria

The expression of keratin intermediate filament proteins is a hallmark of epithelial differentiation. Keratins are subdivided into type I and type II proteins that form obligate heteropolymers in a cell type-, differentiation-, and tissue-specific manner. Simple epithelia, as found in liver, pancreas, and intestine, express the keratin pair keratin 8 and 18 or 19, whereas the stratified epithelium of the skin, the epidermis, expresses a more complex pattern of different keratin pairs depending on the epidermal layer and the status of the keratinocytes [98]. Patients harboring mutations in keratins 5 and 14, the major keratins in the basal layer of the epidermis, develop epidermolysis bullosa simplex (EBS). EBS is characterized by severe blistering due to impaired mechanical stress resilience [99]. Mitochondria of these keratinocytes are abnormally concentrated around the nucleus [46,100].

The lipid composition of keratin-depleted keratinocytes has been shown to be considerably altered [47]. The nonbilayer-forming lipids cardiolipin and phosphatidyl ethanolamine, which are produced in mitochondria, are elevated. The precursors for both lipids are imported from the ER through mitochondria-associated membranes (MAMs) [82,83,101]. An interesting observation in this context is the finding that the keratin-binding protein trichoplein/mitostatin has been shown to function as a regulator of ER-mitochondrium tethering [102,103]. This may be the reason why mitochondrial lipid homeostasis is severely perturbed in the absence of keratins and may also explain the other observed alterations in mitochondrial structure and function [47,104,105].

When livers of keratin 8 knock-out mice were compared to wild type controls changes in mitochondrial morphology were readily detectable. Mitochondria were significantly smaller and irregularly distributed [106]. One possible mechanism by which the distribution of mitochondria is modulated is the formation of a complex consisting of Pirh2 and keratins 8 and 18. Pirh2 is a RING-H2-type ubiquitin E3 ligase, that targets p53 for degradation and was shown to regulate intermediate filament organization [107,108]. Knock-down of either keratin 8/18 or Pirh2 leads to an abnormal juxtanuclear clustering of mitochondria as well as enhanced apoptosis. Expression of the mutant keratin 18 R89C in liver epithelial cells leads to mitochondrial fragmentation, a hallmark of apoptosis [48,109]. 

The influence of keratin-mediated mitochondrial changes also extends to alterations of mitochondrial hexokinase expression [110]. Keratin-deficient hepatocytes display increased levels of mitochondrial hexokinase leading to increased glycogen content. A possible explanation is that loss of keratins releases PKC which is bound through RACK1 [111]. The released PKC may then phosphorylate VDAC. Phosphorylated VDAC in turn recruits hexokinase [112].

## 3. Conclusions and Future Directions

**Figure 2:**
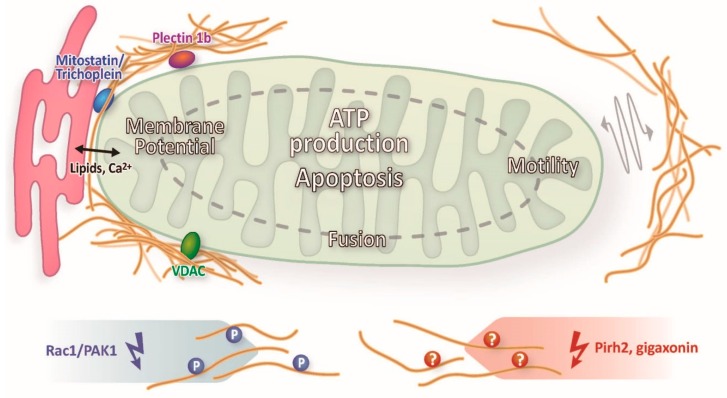
Schematic synopsis of different modes of interaction between intermediate filaments and mitochondria and their consequences as described in this review.

Taken together, convincing evidence has accumulated over the years that intermediate filaments influence the motility, shape and, more importantly, function of mitochondria in different cell types. The scheme in Figure 2 summarizes the described modes of interaction of intermediate filaments and mitochondria and their consequences. Yet, an overall concept of the mechanistic details is still lacking. 

For example, we would like to know whether intermediate filament proteins other than vimentin bind directly to mitochondria. If so, what are the mitochondrial binding sites? Are they mitochondrial-associated membrane components such as VDAC? Are other molecules involved such as plectin and trichoplein/mitostatin? This scenario would provide an explanation how intermediate filaments affect lipid metabolism and Ca^2+^ signaling. In addition, intermediate filaments could control the opening of the mitochondrial permeability transition pore through the same or other interactions and thereby affect apoptosis. In the same vein, it has been suggested recently that mitochondria-associated membranes function as hubs for neurodegeneration, notably in Charcot-Marie-Tooth disease [113].

One would also like to know more about the details of mitochondrial motility control through intermediate filaments and the resulting metabolic alterations. Do intermediate filaments merely provide an inert 3D scaffold or do they represent adjustable docking stations? How do these interactions affect mitochondrial shape? What role do posttranslational intermediate filament protein modifications play in this context [114]?

What role does intermediate filament-dependent subcellular localization of mitochondria play for cellular function? Obvious examples include the local production and availability of ATP in striated muscle cells, neuronal synapses, or migrating cells [60,97,115,116,117]. The relevance may well extend to multiple disease situations such as metastatic tumor cell migration [118,119].

## Figures and Tables

**Table 1:** List of mitochondrial phenotypes observed in cells and tissues harboring intermediate filament network modifications.

Intermediate Filament Protein	Modification	Cell Type	Mitochondrial Phenotype	Reference
NF-L	Knock-Out	Neurons	Decreased length and fusion rate, increased motility	[40]
Peripherin	Overexpression	NFL knock-out neurons	Increased retrograde transport of mitochondria	[41]
Vimentin	Knock-Out	Fibroblasts	Decreased membrane potential, altered distribution, increased motility	[42,43]
Vimentin	Knock-Down	Cos7 cell line	Fragmentation, altered distribution	[44]
Desmin	Knock-Out	Cardiac and skeletal muscle	Abnormal shape and positioning, decreased maximal respiration rate, decreased oxygen consumption	[45]
Keratin 5	P24L mutation	Epidermis	Intracellular clustering	[46]
Keratin	Knock-Out	Epidermis	Altered lipid composition and activity	[47]
Keratin 18	R89C mutation	Liver-derived cell lines	Fragmentation	[48]
Keratin 19	Knock-Out	Muscle	Mitochondrial disorganization	[49]
